# Genetic Evidence Linking Lipoprotein(a) to Cardiovascular Disease and the Potential Role of Aspirin: A Mendelian Randomization Study

**DOI:** 10.31083/RCM39322

**Published:** 2025-10-30

**Authors:** Jiangshan Tan, Wei Xu, Song Hu, Jingyang Wang, Lulu Wang, Jun Zhu, Yanmin Yang

**Affiliations:** ^1^Department of Cardiology, State Key Laboratory of Cardiovascular Disease, National Center for Cardiovascular Diseases, National Clinical Research Center of Cardiovascular Diseases, Fuwai Hospital, Chinese Academy of Medical Sciences and Peking Union Medical College, 100037 Beijing, China; ^2^Heart Failure Center, Department of Cardiology, State Key Laboratory of Cardiovascular Disease, National Center for Cardiovascular Diseases, National Clinical Research Center of Cardiovascular Diseases, Fuwai Hospital, Chinese Academy of Medical Sciences and Peking Union Medical College, 100037 Beijing, China; ^3^Emergency Center, Department of Cardiology, State Key Laboratory of Cardiovascular Disease, National Center for Cardiovascular Diseases, National Clinical Research Center of Cardiovascular Diseases, Fuwai Hospital, Chinese Academy of Medical Sciences and Peking Union Medical College, 100037 Beijing, China

**Keywords:** lipoprotein(a), cardiovascular disease, Mendelian randomization

## Abstract

**Background:**

Many studies have revealed the observational associations between lipoprotein(a) (Lp(a)) concentrations and the incidence of cardiovascular diseases (CVDs). However, the causal associations remain unclear.

**Methods:**

Public summary data were analyzed using a Mendelian randomization (MR) design to assess the causal associations between Lp(a) levels and risks of nine CVDs and evaluate the potential impact of aspirin on Lp(a) levels. The principal analysis was conducted employing the random-effects inverse-variance weighted (IVW) method. Furthermore, the weighted median and MR-Egger approaches were used as the sensitivity analysis. Additionally, the significantly associated single nucleotide polymorphisms (SNPs) in salicylic acid (INTERVAL and EPIC-Norfolk, n = 14,149) were chosen to assess the potential effects of aspirin on lowering Lp(a) levels.

**Results:**

The IVW analysis showed that the per standard deviation (SD) increment in Lp(a) level was causally associated with a higher risk of coronary artery disease (odds ratio (OR), 1.237; 95% confidence interval (CI), 1.173–1.303), atrial fibrillation (OR, 1.030; 95% CI, 1.011–1.050), heart failure (OR, 1.074; 95% CI, 1.053–1.096), hypertension (OR, 1.006; 95% CI, 1.004–1.008), and peripheral artery disease (OR, 1.001; 95% CI, 1.001–1.001) (all *p* < 0.001). The investigation did not reveal any significant heterogeneities or instances of horizontal pleiotropy. Furthermore, for each SD increase in salicylic acid concentration, there was a corresponding 5.4% reduction in Lp(a) levels (OR: 0.946, 95% CI: 0.900–0.993; *p* = 0.022).

**Conclusions:**

A causal nexus was discerned between Lp(a) levels and an increased risk of conditions including coronary artery disease, atrial fibrillation, heart failure, hypertension, and peripheral artery disease. Furthermore, administering aspirin may be a potential therapeutic to reduce these CVD risks among individuals with elevated Lp(a) levels.

## 1. Introduction

Cardiovascular diseases (CVDs) stand as the predominant agents of morbidity and mortality worldwide, bearing the principal burden upon global health [1]. A significant number of cardiovascular risk factors have been recognized up to date and used for predicting outcomes and risk stratification in CVDs. Lipoprotein(a) [Lp(a)] is a liver-derived lipoprotein first identified by Kåre Berg in 1963 [2]. In contemporary discourse, Lp(a) has emerged as a compelling novel risk factor for cardiovascular conditions [3, 4, 5, 6, 7]. Substantial evidence has established that Lp(a) contributes to the pathogenesis of atherosclerosis, vascular calcification, inflammation, and thrombosis [7]. Numerous observational studies have posited a robust correlation between Lp(a) levels and the incidence and prognosis of CVDs [4, 8, 9, 10]. However, these results are predicated on observational data, which are susceptible to confounding variables and the possibility of reverse causation. Prior investigations have probed the causal impact of Lp(a) concentrations on the risk of coronary artery disease (CAD) and peripheral artery disease (PAD) [11, 12, 13]. Notwithstanding, the data for these studies were aggregated from identical cohorts, presenting a complete overlap in samples, which might inflate the perceived causal influence of Lp(a) on the aforementioned conditions. Consequently, the extent of the causal association of Lp(a) with a broad spectrum of CVDs has not been definitively established.

While a definitive pharmacological therapy to reduce Lp(a) levels is still lacking, multiple targeted therapies (e.g., antisense oligonucleotides, siRNAs) are under investigation in clinical trials [14]. Given the structure of oxidized phospholipid components and apolipoprotein(a), it is hypothesized that Lp(a) may facilitate platelet aggregation [15, 16]. Additionally, aspirin has been shown to reduce the production of Lp(a) by inhibiting the expression of apo(a) mRNA in the liver, a process that may not rely on cyclooxygenase-1 [17, 18]. Consequently, it is proposed that individuals with elevated Lp(a) levels may benefit from aspirin therapy [19]. Some studies have suggested that individuals with elevated Lp(a) levels may derive cardiovascular benefit from aspirin therapy, even in the absence of established cardiovascular disease. These findings, however, are based on observational data and genetic subgroups, and their generalizability remains to be clarified [20, 21, 22].

Mendelian randomization (MR) constitutes an innovative methodological approach that employs genetic markers to determine the existence of a causal relationship between a putative risk factor and diseases of interest. Owing to the random inheritance and lifelong stability of genetic variants, MR is less susceptible to confounding factors and reverse causality, thereby serving as a surrogate for randomized clinical trials [23, 24, 25].

The present study was designed to elucidate the role of Lp(a) levels in nine CVDs, including CAD, atrial fibrillation (AF), heart failure (HF), pulmonary embolism, deep vein thrombosis, hypertension, rheumatic and non-rheumatic valve diseases, and PAD. Furthermore, it sought to assess the influence of aspirin on Lp(a) concentrations.

## 2. Method

### 2.1 Overall Study Design 

**Fig. 1.**
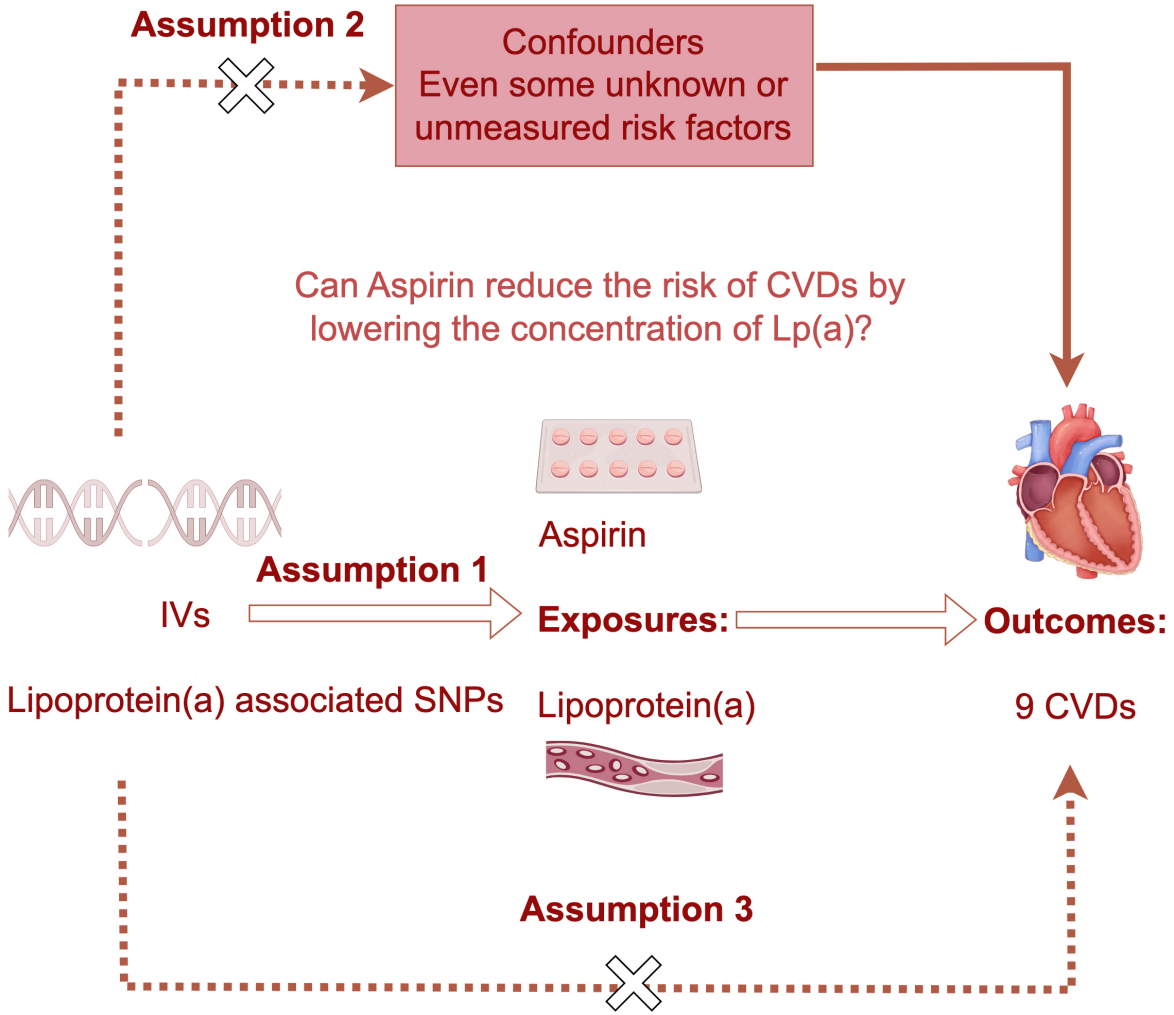
**The schematic overview of the two separate one-directional Mendelian randomization (MR) and mediation analyses**. This study involves two separate one-directional Mendelian randomization and mediation analyses. Single nucleotide polymorphisms associated with lipoprotein(a) levels or Aspirin (salicylic acid) were selected, and the MR analysis was carried out. The inherent randomness and independent assortment of alleles during meiosis endow MR with a potent capacity to ascertain causal relationships, devoid of the biases typical of observational study designs. The dashed arrows denote the lack of significant association between the two variables, and hollow arrows denote the directionality of Mendelian randomization analysis. IVs, instrumental variables; CVDs, cardiovascular diseases; Lp(a), lipoprotein(a); SNPs, single nucleotide polymorphisms.

The overarching architecture of this investigation incorporated a two-sample MR framework to evaluate the causative linkage between Lp(a) concentrations and the risk of nine cardiovascular diseases, utilizing publicly accessible summary datasets [26, 27] (Fig. 1). Additionally, we performed a separate two-sample MR analysis to evaluate the potential causal effect of genetically predicted salicylic acid (SA), used as a proxy for aspirin exposure, on Lp(a) levels. This MR study used publicly available genome-wide association studies (GWAS) summary statistics, all of which had previously received appropriate ethical approval in their original studies.

### 2.2 Data Sources

#### 2.2.1 Exposure: Lp(a) and Aspirin

Within this MR framework, single nucleotide polymorphisms (SNPs) from GWAS were employed as instrumental variables (IVs). The aggregate data for Lp(a) levels were sourced from the Precocious Coronary Artery Disease (PROCARDIS) Consortium, which concluded with a cohort comprising 3145 affected individuals and 3352 control subjects. Besides, these SNPs were reconfirmed in another three independent populations, which included 4846 cases and 4594 control subjects [12]. SA is the active form of the aspirin metabolic pathway and the levels of SA can be supplemented by the deacetylation of aspirin [28]. Thus, we chose the GWAS significant SNPs of SA [29] (from the INTERVAL and EPIC-Norfolk cohorts, with a sample size of 14,149) to examine the causal impact of aspirin on Lp(a) levels. The comprehensive data can be found in Table 1, accessible via the GWAS database at https://gwas.mrcieu.ac.uk/datasets.

**Table 1.** **The attributes of the genome-wide association studies concerning lipoprotein(a) levels and the risk of nine cardiovascular diseases**.

Exposure/Outcomes	No. of controls	No. of cases	Sample size	Year of publication	Number of SNPs	Build	Study population
Lipoprotein(a) levels	-	-	15,937	2009	48,742	HG19/GRCh37	European
Salicylic acid	-	-	14,149	2021	-	HG19/GRCh37	European
Coronary artery disease	424,528	122,733	547,261	2017	7,934,254	HG19/GRCh45	European
Atrial fibrillation	970,216	60,620	1,030,836	2018	33,519,037	HG19/GRCh46	European
Heart failure	930,014	47,309	977,323	2020	7,773,021	HG19/GRCh47	European
Rheumatic valve diseases	218,219	404	218,623	2021	16,380,466	HG19/GRCh48	European
Deep vein thrombosis	453,692	9241	462,933	2018	9,851,867	HG19/GRCh49	European
Hypertension	408,652	54,358	463,010	2018	9,851,867	HG19/GRCh50	European
Pulmonary embolism	461,164	1846	463,010	2018	9,851,867	HG19/GRCh51	European
Non-rheumatic valve diseases	359,588	1606	361,194	2018	10,080,950	HG19/GRCh52	European
Peripheral artery disease	359,964	1230	361,194	2018	9,637,467	HG19/GRCh53	European

SNPs, single nucleotide polymorphisms. All F-statistics for the genetic instruments used in the salicylic acid GWAS exceed 10 (ranging from 30 to 870), indicating that the instruments are robust and have sufficient strength to minimize the potential for weak instrument bias in Mendelian randomization analyses.

#### 2.2.2 Selection Criteria of Instrument Variants

SNPs were selected as IVs according to the following criteria [30, 31]: (1) The IVs demonstrated an association with Lp(a) that surpassed the threshold of genome-wide significance (*p* < 5 × 10^-8^), fulfilling the primary assumption of MR. (2) These IVs for Lp(a) were selected for their independence from one another, adhering to a threshold of *R*^2^ <0.001 and a window size of 10,000 kb to meet the secondary and tertiary assumption of MR. (3) The robustness of the IVs’ association with Lp(a) was confirmed by an F-statistic exceeding 10, with the *F*-statistic being calculated as (β*/SE*)^2^.

### 2.3 Study Outcome: Cardiovascular Diseases

The aggregate data pertinent to CVDs were procured from an MR platform, which boasts a repository of 244,724,428,005 genetic correlations drawn from 42,334 GWAS summary datasets. To examine the causative links between Lp(a) levels and a spectrum of cardiovascular outcomes, an extensive array of CVDs was incorporated into the current MR analysis. This encompassed AF, CAD, deep vein thrombosis of lower extremities, HF, hypertension, PAD, pulmonary embolism, rheumatic valve diseases, and non-rheumatic valve diseases [32]. The cardiovascular outcomes were defined based on clinical criteria from the original GWAS studies. If there are multiple GWASs finished in one disease, the GWAS with a maximum sample size and the most recent published would be chosen. The detailed information on included GWAS has been shown in Table 1.

### 2.4 Statistical Analysis

Information about individualized data was not available contained in the MR platform. Therefore, by using the summary data of published GWAS, the present MR study was performed to evaluate the causal effect of Lp(a) levels on CVDs (Fig. 1), as described in our previous published studies [33].

To ensure reliability, different methods were performed to determine the causal effect based on the degree of heterogeneity. The random-effects inverse-variance weighted (IVW) was performed in the main analysis [34]. Besides, both the weighted median approach [35] and the MR-Egger method [36] were performed in the sensitivity analysis. All three methods are based on the degree of heterogeneity and the consistency of IVW, weighted median and MR-Egger can help to judge the reliability of the present MR [37, 38]. The GWAS summary statistics for both Lp(a) and SA levels were standardized to standard deviation (SD) units. The causal effects were expressed as odds ratios (ORs) with 95% confidence intervals (CIs), representing the change in outcome risk per 1-SD increase in genetically predicted Lp(a) level, or change in Lp(a) level per 1-SD increase in genetically predicted SA level. Besides, modified Cochran *Q* statistics and MR pleiotropic tests were performed to test the potential heterogeneity and horizontal pleiotropy.

A two-tailed significance threshold of *p* < 0.05 was uniformly applied across all statistical tests, with adjustments made via Bonferroni correction. Consequently, associations manifesting *p*-values < 0.006 (α = 0.05/9 outcomes) [39] were deemed to denote a significant causal effect. Associations presenting with *p*-values ranging from 0.006 to 0.05 were indicative of a plausible effect of Lp(a) on cardiovascular disease risk, albeit warranting further corroboration [39]. All statistical analyses were completed using R software (version 4.0.3, R Foundation for Statistical Computing, Vienna, Austria) and the corresponding MR software packages, including usethis version 2.2.3, devtools version 2.4.5, TwoSampleMR version 0.6.3, MendelR version 9.2.37 and MRPRESSO version 1.0 [40, 41]. The study protocol and details were not pre-registered in any websites.

## 3. Results

### 3.1 Genetic IVs for Lp(a) Levels

As delineated in Table 1, the current MR study incorporated ten genome-wide association studies—comprising one GWAS about Lp(a) levels and nine GWAS concerning various CVDs—all of which were conducted within the European demographic. Moreover, nine independent genetic variants for Lp(a) levels, adhering to a linkage disequilibrium threshold of *R*^2^ <0.001 (as detailed in Table 2), were identified and employed as instrumental variables on the strength of a GWAS significance level of *p* < 5 × 10^-8^. The F-statistics for the genetic instruments in the salicylic acid GWAS ranged from 30 to 870, indicating strong instrument strength and reducing the risk of weak instrument bias (Table 1).

**Table 2.** **The compendium of genetic instruments for lipoprotein(a) levels, enumerated by individual instrumental single nucleotide polymorphisms, each meeting the genome-wide association significance criterion (*p* < 5 × 10^-8^) and linkage disequilibrium threshold (R^2^ <0.001)**.

Chr.	Position	beta	se	*p* value	SNP	EA	OA	EAF
6	161010118	1.18	0.04	3.60 × 10^–⁢166^	rs10455872	G	A	0.07
6	160961137	1.27	0.08	5.90 × 10^–⁢51^	rs3798220	C	T	0.02
6	160963230	0.50	0.04	5.90 × 10^–⁢28^	rs11751605	C	T	0.16
6	161069941	0.32	0.04	1.80 × 10^–⁢17^	rs10945682	G	A	0.64
6	160960359	0.43	0.05	1.60 × 10^–⁢16^	rs6919346	C	T	0.83
6	160953035	0.30	0.04	1.50 × 10^–⁢14^	rs3127596	G	A	0.30
6	160969738	0.27	0.04	3.40 × 10^–⁢13^	rs10755578	G	C	0.48
6	160998148	0.28	0.05	2.00 × 10^–⁢9^	rs3798221	G	T	0.81
6	160980330	0.22	0.04	2.70 × 10^–⁢9^	rs6415084	T	C	0.49

Chr., indicates chromosome; EA, effect allele; OA, other allele; EAF, effect allele frequency.

### 3.2 Effects of Lp(a) Levels on Nine Cardiovascular Diseases

**Fig. 2.**
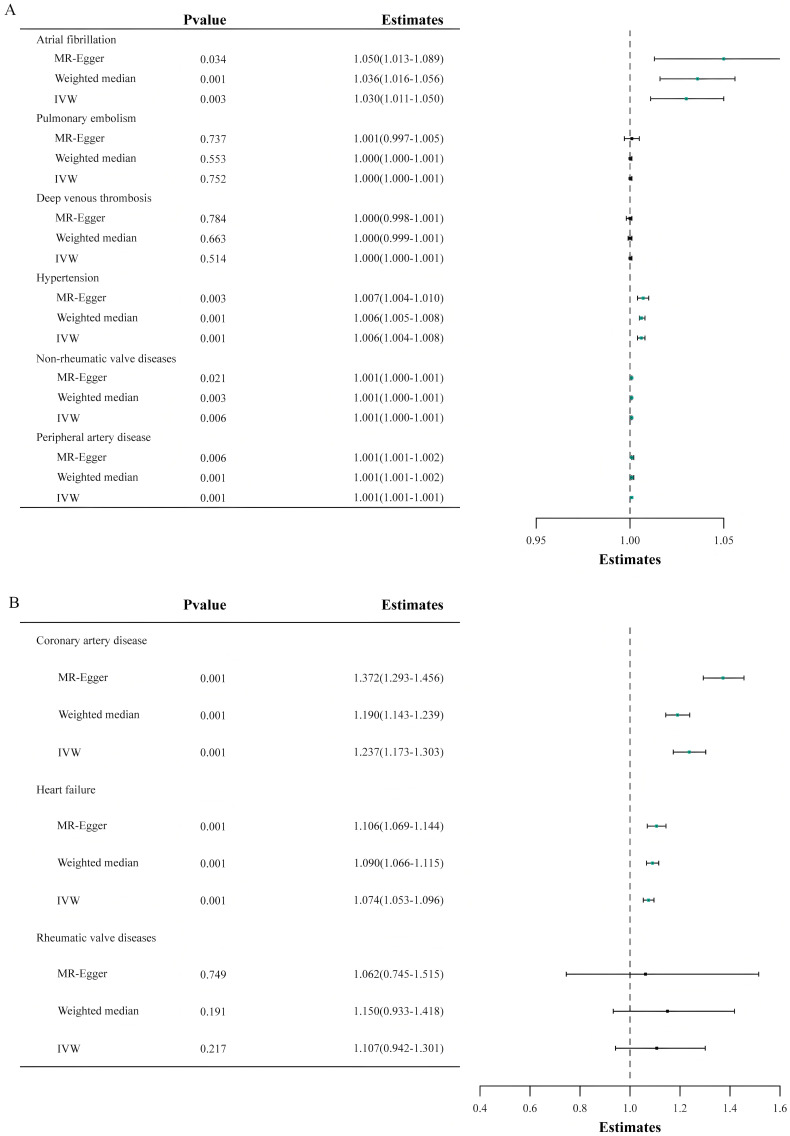
**The outcomes of the Mendelian randomization analysis that probes the link between genetically predicted lipoprotein(a) levels and the risk of nine cardiovascular diseases**. The forest plot illustrates the Mendelian randomization estimates using inverse-variance weighted, weighted median, and MR-Egger analysis methods, delineating the association between lipoprotein(a) levels and the risk of various cardiovascular diseases. IVW, inverse-variance weighted; MR, Mendelian randomization. (A,B) both present results from Mendelian randomization analyses. Because the x-axis ranges differ between the two sets of results, they are shown separately as (A,B) for clarity.

Fig. 2 delineates the impact of genetically inferred Lp(a) levels on the susceptibility to nine CVDs. In the principal analysis, MR analysis using the IVW method indicated that each one SD increase in genetically predicted Lp(a) levels was associated with a 23.7% increased risk of CAD (OR = 1.237, 95% CI: 1.173–1.303, *p* < 0.001), a 3.0% increased risk of AF (OR = 1.030, 95% CI: 1.011–1.050, *p* < 0.001), a 7.4% increased risk of HF (OR = 1.074, 95% CI: 1.053–1.096, *p* < 0.001), a 0.6% increased risk of hypertension (OR = 1.006, 95% CI: 1.004–1.008, *p* < 0.001), and a 0.1% increased risk of peripheral artery disease (OR = 1.001, 95% CI: 1.001–1.001, *p* < 0.001).

Furthermore, a potential causal link between Lp(a) levels and non-rheumatic valve diseases was suggested with an OR of 1.000 (95% CI, 1.000–1.001; *p* = 0.006). Nonetheless, no causal relationship was found between Lp(a) levels and deep vein thrombosis of lower extremities, pulmonary embolism, or rheumatic valve diseases (*p* > 0.05). Notably, the ORs for hypertension, peripheral artery disease, and non-rheumatic valve diseases were found to be relatively minor.

### 3.3 Effects of Aspirin (Salicylic Acid) on Lp(a) Levels 

**Fig. 3.**
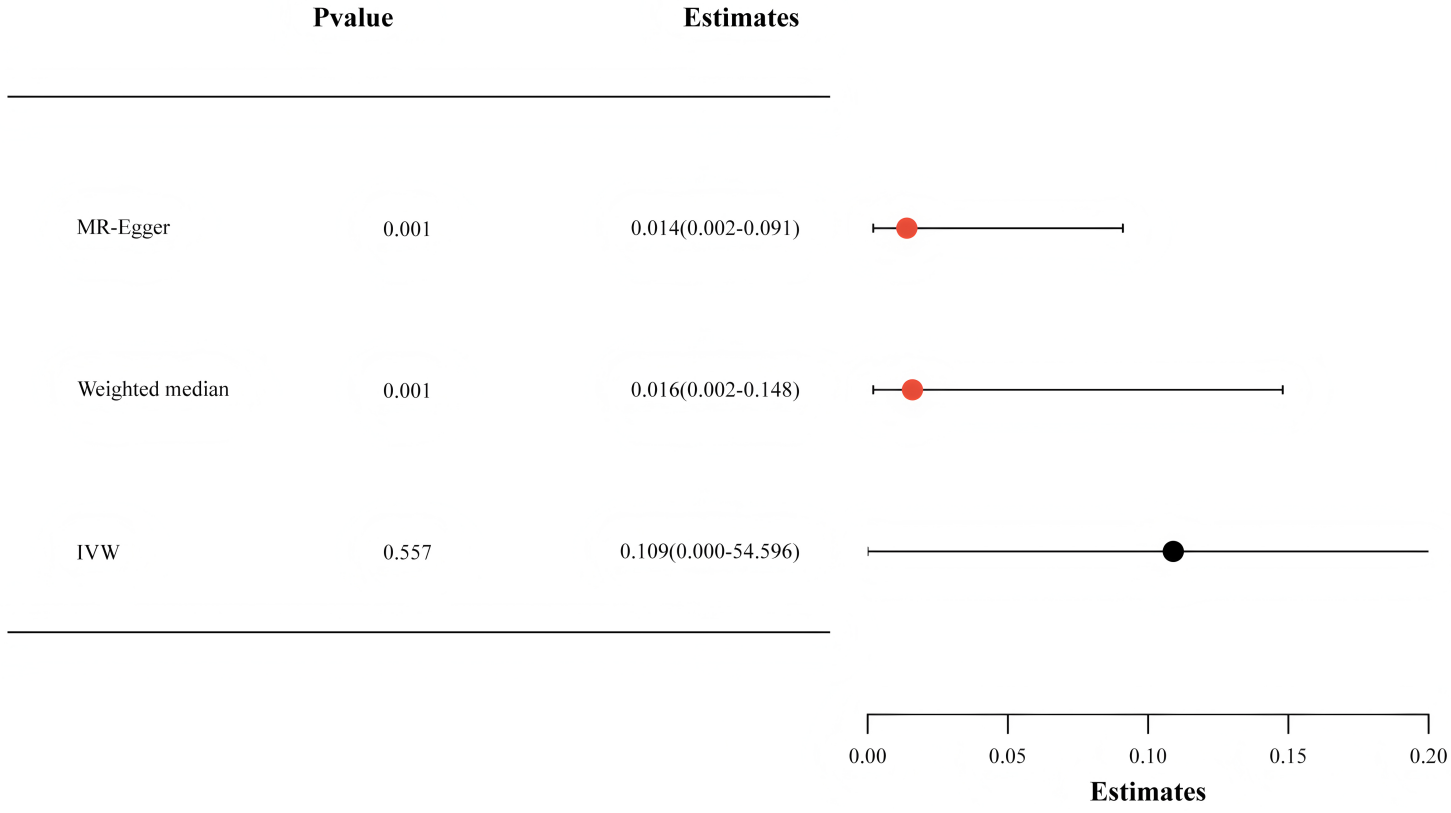
**The findings from the Mendelian randomization analysis exploring the relationship between salicylic acid and lipoprotein(a) levels**. IVW, inverse-variance weighted.

SA, the active form of the aspirin metabolic pathway, can be supplemented by the deacetylation of aspirin [28]. Therefore, we chose SA-associated SNPs for the MR analysis. Our investigation revealed that with each SD increase in SA concentration, there was a corresponding reduction in Lp(a) levels by 5.4% (OR: 0.946, 95% CI: 0.900–0.993, *p* = 0.022) (Fig. 3).

### 3.4 Sensitivity Analysis for MR Analysis

To ascertain the reliability of our methodology, we employed various validation methods, such as the weighted median and MR-Egger analysis. Figs. 2,3 depict that comparable causal estimations emerged from the sensitivity analysis, albeit with diminished precision.

### 3.5 Analysis of Heterogeneity and Horizontal Pleiotropy

In the current MR analysis, the modified Cochran *Q* statistic indicated an absence of significant heterogeneity (*p* > 0.05). Furthermore, the MR pleiotropic tests did not demonstrate any horizontal pleiotropy (intercept *p*-value > 0.05), implying that the results of our study are both robust and reliable.

## 4. Discussion

In this MR study, the findings revealed a causative association between elevated Lp(a) levels and an increased risk of CAD, AF, HF, hypertension, and PAD. Notably, genetically predicted higher SA levels were associated with lower Lp(a) levels in our analysis. While this observation may support a potential role for aspirin in modulating Lp(a)-related cardiovascular risk, this hypothesis requires validation in prospective clinical studies.

Growing evidence from observational studies supported the role of Lp(a) in atherogenesis and thrombosis [42, 43, 44]. The Copenhagen City Heart Study from the general Danish population examined 9330 individuals for 10 years and found that elevated Lp(a) levels were related to an increased risk of myocardial infarction (MI) [42]. A meta-analysis of 36 prospective studies summarized a total of 126,634 individuals indicated that there was a continuous and robust association between Lp(a) concentration and the risk of CAD [43]. Another analysis from the UK Biobank database enrolled 460,506 individuals demonstrated a linear relationship between Lp(a) and the risk of CAD during a median of 11.2 years follow-up [44]. Two large genetic epidemiological studies causally revealed Lp(a) concentration in the CAD population [12, 13]. Consistent with these findings, our MR analysis supports the causal relationship between elevated Lp(a) levels and an increased risk of CAD. Epidemiologic studies indicated only an extremely high Lp(a) concentration was correlated with a slightly increased risk of venous thromboembolism [45]. However, we did not observe a causal association between Lp(a) levels and deep vein thrombosis of lower extremities or pulmonary embolism in this study, which is consistent with a previous MR analysis [46].

Studies of evaluating the impact of Lp(a) levels on AF are limited and the relationship between the two has not been evaluated effectively. A community-based cohort study with a median follow-up of 13.9 years indicated that elevated Lp(a) levels were not correlated with the risk of AF [47]. However, this observational cohort study did not include extremely high Lp(a) concentrations and excluded individuals aged over 65 years old; the results may be influenced by potential confounders and sample selection bias. In contrast, a recent observational study conducted in the UK biobank database suggested each 50 nmol/L elevation in Lp(a) was correlated with an increased risk of AF occurrence [Hazard ratio (HR): 1.03; 95% CI: 1.02–1.04; *p* < 0.05] [48]. Consistent with our study, the MR analyses revealed that Lp(a) may be a causal mediator in the incidence of AF [48]. A large prospective cohort from Europe demonstrated that Lp(a) levels were significantly associated with the occurrence of peripheral artery disease [49]. Our results by MR analysis further complemented the causal association between Lp(a) levels and the risk of PAD.

The Multi-Ethnic Study of Atherosclerosis (MESA) study, which included 6809 participants, suggested that elevated Lp(a) levels were associated with a higher risk of HF [Lp(a) ≥50 mg/dL; HR, 1.87; *p* = 0.006] in the white population, which is consistence with our results [50]. Similarly, although limited data exists on the role of Lp(a) in hypertension, a cohort study found that 30% of hypertensive individuals had evaluated Lp(a) levels [51]. Despite these findings, observational studies alone cannot clarify the precise association between Lp(a) levels and the incidence of HF and hypertension. Our study is the first to demonstrate a causal relationship between elevated Lp(a) levels and the risk of both HF and hypertension.

The question of whether targeted Lp(a) reduction therapies can mitigate cardiovascular event risks has ignited considerable interest. However, the current challenge is the absence of specific therapies aimed at lowering Lp(a) levels. A randomized controlled trial (RCT) by Lacaze *et al*. [22] examined the effect of aspirin on subjects with genotypes related to high plasma Lp(a), revealing that aspirin diminished the incidence of major adverse cardiovascular events in individuals over 70 years old. However, this RCT could not eliminate the potential impact of other variables, such as statin therapy, which may elevate Lp(a) levels. Furthermore, direct measurements of Lp(a) levels were not taken, leaving the specific influence of aspirin on Lp(a) levels undetermined. Notably, Bhatia *et al*. [52] conducted an observational study using the MESA cohort and demonstrated that aspirin use was associated with a significantly lower risk of coronary heart disease events in individuals with Lp(a) >50 mg/dL, suggesting a potential benefit of aspirin for primary prevention in this population. However, their study was limited by the inherent drawbacks of observational studies, such as the potential bias, and the definition of aspirin use was based on patient self-reports. In contrast, our MR study leveraged genetic variants to minimize confounding and reverse causality, providing supportive causal inference regarding the inverse association between SA levels and Lp(a) concentrations, Increasing the possibility that aspirin may lead to a decrease in Lp(a). These studies underscore the urgent need for RCT to clarify the role of aspirin in managing cardiovascular risk associated with elevated Lp(a). To date, the mechanism by which aspirin may reduce Lp(a) levels remains unclear. Previous in vitro studies have shown that aspirin and sodium salicylate can inhibit the transcription and mRNA expression of apo(a) gene in human hepatocytes, thereby reducing Lp(a) synthesis, while the non-selective cyclooxygenase inhibitor indomethacin is ineffective, indicating that this process is independent of cyclooxygenase inhibition [17, 18]. These results are consistent with Mendelian randomization analyses that we found a negative correlation between SA levels and Lp(a) concentrations. Further studies are needed to clarify this mechanism and its clinical implications.

The principal merit of this investigation lies in the application of the MR methodology to establish a causal relationship between Lp(a) levels and the risks of nine CVDs within a relatively homogeneous population, distinctly without overlap in the study cohort. Our findings bolster the evidence for a causal link between Lp(a) and cardiovascular risk, aligning with the recent declaration by the European Atherosclerosis Society consensus [6].

Adhering to Mendel’s Second Law, which posits the independent assortment of alleles, each heritable trait segregates independently during gamete formation. Consequently, in a relatively homogenous population, the random distribution of genotypes amidst potential confounders allows for the inference of causality under conditions akin to those in a RCT [53]. Thus, our MR analysis is poised to circumvent biases that typically arise from confounding factors and reverse causality. Moreover, the stability of Lp(a) concentrations throughout an adult’s life, governed by genetic variation, renders it an exemplary subject for MR analysis [5]. The outcomes of this research might well reflect the lifelong implications of Lp(a) levels on cardiovascular disease risk. This MR study was specifically conducted within a European ancestry population using GWAS data for Lp(a) levels and CVDs, which serves to minimize the impact of bias and confounding variables. Additionally, MR-Egger regression was utilized to ascertain that the SNPs exert their effects on CVDs solely through Lp(a) levels and no directional pleiotropic effects were detected in this MR analysis.

However, it is worth noting that several outcomes in our MR analysis exhibit unusually narrow confidence intervals. This pattern is likely a consequence of the large sample sizes and the small effect sizes commonly observed in GWAS. While narrow confidence intervals suggest high statistical precision, they should be interpreted in the context of the underlying data structure and study design. Specifically, large sample sizes can yield precise estimates even when the magnitude of the effect is modest, which may not always reflect stronger causal relationships. Therefore, these findings should be interpreted with caution and regarded as hypothesis-generating. Further studies—particularly those involving independent cohorts and complementary methodological approaches—are warranted to validate and extend these results.

## 5. Limitations

Several limitations should be acknowledged in this study. Primarily, due to reliance on summary data, individual patient-level information was inaccessible; consequently, it was not feasible to stratify the association between Lp(a) and CVDs by sex and age within this study. Second, the research encompassed solely the European demographic; hence, additional studies are requisite to determine whether these results are applicable to other ethnicities. Third, given the inherent limitations of MR studies, although we used MR-Egger, weighted median, and Cochran *Q* test to detect and reduce pleiotropy and heterogeneity, residual bias for undetected pleiotropy or gene-environment interactions could not be entirely ruled out. Fourth, multiple testing may increase the risk of type I error, thus some statistically significant findings should be interpreted with caution. Fifth, our results suggested that genetically predicted SA levels were inversely correlated with Lp(a), but SA is not unique to aspirin metabolism and lacks the pharmacological effects of aspirin. Sixth, all nine genetic variants used as instruments for Lp(a) are located within the lipoprotein(a) gene locus on chromosome 6. While LD-clumping with an R^2^ threshold of <0.001 was employed to minimize statistical correlation among the variants, it is important to acknowledge that their shared biological context may limit the ability of MR-Egger and related tests to detect horizontal pleiotropy. This potential limitation must be considered when interpreting the results, as the biological effects of these variants within the same locus may introduce confounding factors that obscure the detection of pleiotropy. Therefore, our findings provide indirect evidence for the potential Lp(a)-lowering effects of aspirin, which still require further clinical validation in the future. Finally, the observed odds ratios for hypertension, peripheral artery disease, and non-rheumatic valve diseases were relatively modest, which may be influenced by the large sample size used in this study. Therefore, these findings should be interpreted with caution. Further research is needed to reaffirm the causal links between Lp(a) levels and these three cardiovascular conditions.

## 6. Conclusions

In summary, our MR analysis lends credence to a causal connection between Lp(a) levels and the risk of CVDs including CAD, AF, HF, hypertension, and PAD, suggesting individuals may benefit from reducing Lp(a) levels, which can be expected to be a new target for lowering cardiovascular risk. Furthermore, aspirin may be an effective therapeutic agent to reduce CVD risks in individuals with elevated Lp(a) levels in the future, though this hypothesis warrants prospective clinical validation.

## Availability of Data and Materials

All summary-level GWAS data used in this study are publicly available from the IEU OpenGWAS database (https://gwas.mrcieu.ac.uk/). No individual-level data were used. The analysis code is available upon reasonable request from the corresponding author.

## Supplementary Material

Supplementary material associated with this article can be found, in the online version, at https://doi.org/10.31083/RCM39322.
