# Supplementary material for: Genetic Evidence Linking Lipoprotein(a) to Cardiovascular Disease and the Potential Role of Aspirin: A Mendelian Randomization Study
Source: Rev Cardiovasc Med. 2025 Oct 30;26(10):39322. doi: 10.31083/RCM39322 (PMC12593744; doi:10.31083/RCM39322)
Supplement: Supplementary file 1 [file 2153-8174-26-10-39322-s1.docx]

**STROBE-MR checklist of recommended items to address in reports of Mendelian randomization studies**^1^ ^2^

| **Item No.** | **Section** | **Checklist item** | **Page No.** | **Relevant text from manuscript** |
| --- | --- | --- | --- | --- |
| 1 | **TITLE and ABSTRACT** | Indicate Mendelian randomization (MR) as the study’s design in the title and/or the abstract if that is a main purpose of the study | 1 | **Genetic Evidence Linking Lipoprotein(a) to Cardiovascular Disease and the Potential Role of Aspirin: A Mendelian Randomization Study**  **Abstract**  **Background:** Many studies have revealed the observational associations between lipoprotein(a) [Lp(a)] concentrations and the incidence of cardiovascular diseases (CVDs). However, the causal associations remain unclear.  **Methods:** Mendelian randomization (MR) design was used to assess the causal associations between Lp(a) levels and risks of nine CVDs and evaluate the potential impact of aspirin on Lp(a) levels via public summary data. The principal analysis was conducted employing the random-effects inverse-variance weighted (IVW) method. Furthermore, the weighted median and MR-Egger approaches were used as the sensitivity analysis. Additionally, the significantly associated single-nucleotide polymorphisms (SNPs) of salicylic acid (INTERVAL and EPIC-Norfolk, N = 14,149) were chosen to assess the putative effects of aspirin on lowering the levels of Lp(a).  **Results:** IVW showed that per standard deviation (SD) increment in Lp(a) level was causally associated with a higher risk of coronary artery disease [odds ratio (OR),1.237; 95% conﬁdence interval (CI), 1.173-1.303], atrial fibrillation (OR, 1.030; 95% CI, 1.011-1.050), heart failure (OR, 1.074; 95% CI, 1.053-1.096), hypertension (OR, 1.006; 95% CI, 1.004-1.008), peripheral artery disease (OR, 1.001; 95% CI, 1.001-1.001) (all *p*<0.001). The investigation did not reveal any significant heterogeneities or instances of horizontal pleiotropy. Furthermore, for each SD increase in salicylic acid concentration, there was a corresponding reduction in Lp(a) levels by 5.4% (OR: 0.946, 95% CI: 0.900-0.993, *p*=0.022).  **Conclusions:** A causal nexus was discerned between Lp(a) levels and the increased risk of conditions such as coronary artery disease, atrial fibrillation, heart failure, hypertension, and peripheral artery disease. Furthermore, aspirin may be a potential therapeutic to reduce these CVD risks among individuals with elevated Lp(a) levels.    **Keywords:** Lipoprotein (a), Cardiovascular disease, Mendelian randomization |
|  | **INTRODUCTION** |  |  |  |
| 2 | **Background** | Explain the scientific background and rationale for the reported study. What is the exposure? Is a potential causal relationship between exposure and outcome plausible? Justify why MR is a helpful method to address the study question | 1-2 | Cardiovascular diseases (CVDs) stand as the predominant agents of morbidity and mortality worldwide, bearing the principal burden upon global health [1]. A significant number of cardiovascular risk factors have been recognized up to date and used for predicting outcomes and risk stratification in CVDs. Lipoprotein(a) [lp(a)] is a liver-derived lipoprotein first identified by Kåre Berg in 1963 [2]. In contemporary discourse, Lp(a) has emerged as a compelling novel risk factor for cardiovascular conditions [3-7]. Substantial evidence has established that Lp(a) contributes to the pathogenesis of atherosclerosis, vascular calcification, inflammation, and thrombosis[7]. Numerous observational studies have posited a robust correlation between Lp(a)levels and the incidence and prognosis of CVDs [4, 8-10]. However, these results are predicated on observational data, which are susceptible to confounding variables and the possibility of reverse causation. Prior investigations have probed the causal impact of Lp(a) concentrations on the risk of coronary artery disease (CAD) and peripheral artery disease (PAD) [11-13]. Notwithstanding, the data for these studies were aggregated from identical cohorts, presenting a complete overlap in samples, which might inflate the perceived causal influence of Lp(a) on the aforementioned conditions. Consequently, the extent of the causal association of Lp(a) with a broad spectrum of CVDs has not been definitively established.  While a definitive pharmacological therapy to reduce Lp(a) levels is still lacking, with the phase 2 clinical trial for Lepadisiran, a short interfering RNA targeting a component of Lp(a)currently ongoing and its safety and efficacy under evaluation [14]. Given the structure of oxidized phospholipid components and apolipoprotein(a), it is hypothesized that Lp(a) may facilitate platelet aggregation [15, 16]. Additionally, aspirin has been shown to reduce the production of Lp(a) by inhibiting the expression of apo(a) mRNA in the liver, a process that may not rely on cyclooxygenase-1[17, 18]. Consequently, it is proposed that individuals with elevated Lp (a) levels may benefit from aspirin therapy [19]. Greco et al. pointed out that in individuals with Lp(a) levels > 50 mg/dL and no established cardiovascular disease, aspirin use was significantly associated with a reduction in cardiovascular events, which contrasts with the neutral or unfavorable outcomes of aspirin in primary prevention among general populations[20]. Razavi et al observed that regular aspirin use was associated with significantly reduced atherosclerotic cardiovascular disease (ASCVD) mortality in adults without ASCVD but with elevated Lp(a) levels, highlighting the potential benefit of aspirin in primary prevention[21]. However, the data supporting a causal link between aspirin use and reduced Lp(a) levels are scant. Lacaze et al. have intimated that low-dose aspirin might offer protective benefits against cardiovascular events in elderly individuals with a genotype of high Lp(a) levels, however, this evidence is circumscribed to the single genetic variant rs3798220[22].  Mendelian randomization (MR) constitutes an innovative methodological approach that employs genetic markers to determine the existence of a causal relationship between a putative risk factor and diseases of interest. Owing to the random inheritance and lifelong stability of genetic variants, MR is less susceptible to confounding factors and reverse causality, thereby serving as a surrogate for randomized clinical trials [23-25]. |
| 3 | **Objectives** | State specific objectives clearly, including pre-specified causal hypotheses (if any). State that MR is a method that, under specific assumptions, intends to estimate causal effects | 2 | The present study was designed to elucidate the role of Lp(a) levels in nine CVDs, including CAD, atrial fibrillation (AF), heart failure, pulmonary embolism (PE), deep vein thrombosis (DVT), hypertension, rheumatic and non-rheumatic valve diseases, and PAD. Furthermore, it sought to assess the influence of aspirin on Lp(a) concentrations. |
|  | **METHODS** |  |  |  |
| 4 | **Study design and data sources** | Present key elements of the study design early in the article. Consider including a table listing sources of data for all phases of the study. For each data source contributing to the analysis, describe the following: |  |  |
|  | a) | Setting: Describe the study design and the underlying population, if possible. Describe the setting, locations, and relevant dates, including periods of recruitment, exposure, follow-up, and data collection, when available. | 2 | **Overall study design**  The overarching architecture of this investigation incorporated a two-sample MR framework to evaluate the causative linkage between Lp(a)concentrations and the risk of nine cardiovascular afflictions, utilizing publicly accessible summary datasets [26, 27] (**Figure 1**). Additionally, we performed a separate two-sample MR analysis to evaluate the potential causal effect of genetically predicted salicylic acid (SA), used as a proxy for aspirin exposure, on Lp(a) levels.The requirement for ethics committee approval was waived in the present MR study, because the ethical approvals have been obtained in institutions based on their previously published studies, respectively. |
|  | b) | Participants: Give the eligibility criteria, and the sources and methods of selection of participants. Report the sample size, and whether any power or sample size calculations were carried out prior to the main analysis | 2 | **Data sources**  **Exposure: Lp(a) and aspirin**  Within this MR framework, single-nucleotide polymorphisms (SNPs) from genome-wide association studies (GWAS) were employed as instrumental variables (IVs). The aggregate data for Lp(a) levels were sourced from the Precocious Coronary Artery Disease (PROCARDIS) Consortium, which concluded with a cohort comprising 3,145 affected individuals and 3,352 control subjects. Besides, these SNPs were reconfirmed in another three independent populations, which included 4846 cases and 4594 control subjects [12]. SA is the active form of the aspirin metabolic pathway and the levels of SA can be supplemented by the deacetylation of aspirin [28]. Thus, we chose the GWAS significant SNPs of SA [29] (from the INTERVAL and EPIC-Norfolk cohorts, with a sample size of 14,149) to examine the causal impact of aspirin on Lp(a) levels. The comprehensive data on Lp(a) can be found in **Table 1**, accessible via the GWAS database at <https://gwas.mrcieu.ac.uk/datasets/ukb-d-30790_raw/>.  **Study outcome: Cardiovascular diseases**  The aggregate data pertinent to CVDs were procured from an MR platform, which boasts a repository of 244,724,428,005 genetic correlations drawn from 42,334 GWAS summary datasets. To examine the causative links between Lp(a) levels and a spectrum of cardiovascular outcomes, an extensive array of CVDs was incorporated into the current MR analysis. This encompassed AF, CAD, DVT of lower extremities, heart failure, hypertension, PAD, PE, rheumatic valve diseases, and non-rheumatic valve diseases [32]. The cardiovascular outcomes were defined based on clinical criteria from the original GWAS studies. If there are multiple GWASs finished in one disease, the GWAS with a maximum sample size and the most recent published would be chosen. The detailed information on included GWAS has been shown in **Table 1**. |
|  | c) | Describe measurement, quality control and selection of genetic variants | 2 | **Selection criteria of instrument variants**  SNPs were selected as IVs according to the following criteria ^[30, 31]^: (1) The IVs demonstrated an association with Lp(a) that surpassed the threshold of genome-wide significance (*p* < 5 × 10^-8), fulfilling the primary assumption of MR. (2) These IVs for Lp(a) were selected for their independence from one another, adhering to a threshold of *R²* < 0.001 and a window size of 10,000 kb to meet the secondary and tertiary assumption of MR. (3) The robustness of the IVs' association with Lp(a) was confirmed by an F-statistic exceeding 10, with the *F*-statistic being calculated as (*β/SE*)^2^. |
|  | d) | For each exposure, outcome, and other relevant variables, describe methods of assessment and diagnostic criteria for diseases | 2 | The cardiovascular outcomes were defined based on clinical criteria from the original GWAS studies. |
|  | e) | Provide details of ethics committee approval and participant informed consent, if relevant | NA | The requirement for ethics committee approval was waived in the present MR study, because the ethical approvals have been obtained in institutions based on their previously published studies, respectively. |
| 5 | **Assumptions** | Explicitly state the three core IV assumptions for the main analysis (relevance, independence and exclusion restriction) as well assumptions for any additional or sensitivity analysis | 2 | **Selection criteria of instrument variants**  SNPs were selected as IVs according to the following criteria ^[30, 31]^: (1) The IVs demonstrated an association with Lp(a) that surpassed the threshold of genome-wide significance (*p* < 5 × 10^-8), fulfilling the primary assumption of MR. (2) These IVs for Lp(a) were selected for their independence from one another, adhering to a threshold of *R²* < 0.001 and a window size of 10,000 kb to meet the secondary and tertiary assumption of MR. (3) The robustness of the IVs' association with Lp(a) was confirmed by an F-statistic exceeding 10, with the *F*-statistic being calculated as (*β/SE*)^2^. |
| 6 | **Statistical methods: main analysis** | Describe statistical methods and statistics used |  |  |
|  | a) | Describe how quantitative variables were handled in the analyses (i.e., scale, units, model) | 2-3 | The GWAS summary statistics for both Lp(a) and SA levels were standardized to standard deviation (SD) units. The causal effects were expressed as odds ratios (ORs) with 95% confidence intervals (CIs), representing the change in outcome risk per 1-SD increase in genetically predicted Lp(a) level, or change in Lp(a) level per 1-SD increase in genetically predicted SA level. |
|  | b) | Describe how genetic variants were handled in the analyses and, if applicable, how their weights were selected | 2 | To ensure reliability, different methods were performed to determine the causal effect based on the degree of heterogeneity. The random-effects inverse-variance weighted (IVW) was performed in the main analysis [34]. Besides, both the weighted median approach [35] and the MR-Egger method [36] were performed in the sensitivity analysis. All three methods are based on the degree of heterogeneity and the consistency of IVW, weighted median and MR-Egger can help to judge the reliability of the present MR [37, 38]. |
|  | c) | Describe the MR estimator (e.g. two-stage least squares, Wald ratio) and related statistics. Detail the included covariates and, in case of two-sample MR, whether the same covariate set was used for adjustment in the two samples | 2 | To ensure reliability, different methods were performed to determine the causal effect based on the degree of heterogeneity. The random-effects inverse-variance weighted (IVW) was performed in the main analysis [34]. Besides, both the weighted median approach [35] and the MR-Egger method [36] were performed in the sensitivity analysis. All three methods are based on the degree of heterogeneity and the consistency of IVW, weighted median and MR-Egger can help to judge the reliability of the present MR [37, 38]. |
|  | d) | Explain how missing data were addressed | - | There is no missing data. |
|  | e) | If applicable, indicate how multiple testing was addressed | 3 | Consequently, associations manifesting *p*-values < 0.006 (*α* = 0.05/9 outcomes) [39] were deemed to denote a significant causal effect. Associations presenting with *p* -values ranging from 0.006 to 0.05 were indicative of a plausible effect of Lp(a) on cardiovascular disease risk, albeit warranting further corroboration [39]. |
| 7 | **Assessment of assumptions** | Describe any methods or prior knowledge used to assess the assumptions or justify their validity | 2 | **Selection criteria of instrument variants**  SNPs were selected as IVs according to the following criteria ^[30, 31]^: (1) The IVs demonstrated an association with Lp(a) that surpassed the threshold of genome-wide significance (*p* < 5 × 10^-8), fulfilling the primary assumption of MR. (2) These IVs for Lp(a) were selected for their independence from one another, adhering to a threshold of *R²* < 0.001 and a window size of 10,000 kb to meet the secondary and tertiary assumption of MR. (3) The robustness of the IVs' association with Lp(a) was confirmed by an F-statistic exceeding 10, with the *F*-statistic being calculated as (*β/SE*)^2^. |
| 8 | **Sensitivity analyses and additional analyses** | Describe any sensitivity analyses or additional analyses performed (e.g. comparison of effect estimates from different approaches, independent replication, bias analytic techniques, validation of instruments, simulations) | 2 | To ensure reliability, different methods were performed to determine the causal effect based on the degree of heterogeneity. The random-effects inverse-variance weighted (IVW) was performed in the main analysis [34]. Besides, both the weighted median approach [35] and the MR-Egger method [36] were performed in the sensitivity analysis. All three methods are based on the degree of heterogeneity and the consistency of IVW, weighted median and MR-Egger can help to judge the reliability of the present MR [37, 38]. |
| 9 | **Software and pre-registration** |  |  |  |
|  | a) | Name statistical software and package(s), including version and settings used | 3 | All statistical analyses were completed using R version 4.0.3 and the corresponding MR software packages, including usethis version 2.2.3, devtools version 2.4.5, TwoSampleMR version 0.6.3, MendelR version 9.2.37 and MRPRESSO version 1.0 [40, 41]. |
|  | b) | State whether the study protocol and details were pre-registered (as well as when and where) | 3-4 | The study protocol and details wasn’t pre-registered in any websites. |
|  | **RESULTS** |  |  |  |
| 10 | **Descriptive data** |  |  |  |
|  | a) | Report the numbers of individuals at each stage of included studies and reasons for exclusion. Consider use of a flow diagram | 4 | **Genetic IVs for Lp (a) levels**  As delineated in **Table 1**, the current MR study incorporated ten genome-wide association studies — comprising one GWAS about Lp(a) levels and nine GWAS concerning various CVDs — all of which were conducted within the European demographic. |
|  | b) | Report summary statistics for phenotypic exposure(s), outcome(s), and other relevant variables (e.g. means, SDs, proportions) | 4 | Moreover, nine independent genetic variants for Lp(a) levels, adhering to a linkage disequilibrium threshold of *R²* < 0.001 (as detailed in **Table 2**), were identified and employed as instrumental variables on the strength of a GWAS significance level of *p* < 5 × 10^-8. |
|  | c) | If the data sources include meta-analyses of previous studies, provide the assessments of heterogeneity across these studies | 5 | **Analysis of heterogeneity and horizontal pleiotropy**  In the current MR analysis, the modified Cochran *Q* statistic indicated an absence of significant heterogeneity (*p* > 0.05). Furthermore, the MR pleiotropic tests did not demonstrate any horizontal pleiotropy (intercept *p*-value > 0.05), implying that the results of our study are both robust and reliable. |
|  | d) | For two-sample MR:  i.  Provide justification of the similarity of the genetic variant-exposure associations between the exposure and outcome samples  ii.  Provide information on the number of individuals who overlap between the exposure and outcome studies | - | - |
| 11 | **Main results** |  |  |  |
|  | a) | Report the associations between genetic variant and exposure, and between genetic variant and outcome, preferably on an interpretable scale | 4 | **Genetic IVs for Lp (a) levels**  As delineated in **Table 1**, the current MR study incorporated ten genome-wide association studies — comprising one GWAS about Lp(a) levels and nine GWAS concerning various CVDs — all of which were conducted within the European demographic. |
|  | b) | Report MR estimates of the relationship between exposure and outcome, and the measures of uncertainty from the MR analysis, on an interpretable scale, such as odds ratio or relative risk per SD difference | 4-5 | **Effects of Lp(a) levels on nine cardiovascular diseases**  **Figure 2** delineates the impact of genetically inferred Lp(a) levels on the susceptibility to nine CVDs. In the principal analysis, MR analysis using the IVW method indicated that each one SD increase in genetically predicted Lp(a) levels was associated with a 23.7% increased risk of CAD (OR = 1.237, 95% CI: 1.173–1.303, *p* < 0.001), a 3.0% increased risk of AF (OR = 1.030, 95% CI: 1.011–1.050, *p* < 0.001), a 7.4% increased risk of heart failure (OR = 1.074, 95% CI: 1.053–1.096, *p* < 0.001), a 0.6% increased risk of hypertension (OR = 1.006, 95% CI: 1.004–1.008, *p* < 0.001), and a 0.1% increased risk of peripheral artery disease (OR = 1.001, 95% CI: 1.001–1.001, *p* < 0.001).  Furthermore, a potential causal link between Lp(a) levels and non-rheumatic valve diseases was suggested with an OR of 1.000 (95% CI, 1.000-1.001; *p* = 0.006). Nonetheless, no causal relationship was found between Lp(a) levels and deep vein thrombosis of lower extremities, pulmonary embolism, or rheumatic valve diseases (*p* > 0.05). Notably, the ORs for hypertension, peripheral artery disease, and non-rheumatic valve diseases were found to be relatively minor.  **Effects of aspirin (salicylic acid) on Lp(a) levels**  SA, the active form of the aspirin metabolic pathway, can be supplemented by the deacetylation of aspirin.[28]. Therefore, we chose SA-associated SNPs for the MR analysis. Our investigation revealed that with each SD increase in SA concentration, there was a corresponding reduction in Lp(a) levels by 5.4% (OR: 0.946, 95% CI: 0.900-0.993,  *p* =0.022) (Figure 3). |
|  | c) | If relevant, consider translating estimates of relative risk into absolute risk for a meaningful time period | 4-5 | As mentioned above. |
|  | d) | Consider plots to visualize results (e.g. forest plot, scatterplot of associations between genetic variants and outcome versus between genetic variants and exposure) | 6-7 | Figure 2 and Figure 3. |
| 12 | **Assessment of assumptions** |  |  |  |
|  | a) | Report the assessment of the validity of the assumptions | 4 | **Genetic IVs for Lp (a) levels**  As delineated in **Table 1**, the current MR study incorporated ten genome-wide association studies — comprising one GWAS about Lp(a) levels and nine GWAS concerning various CVDs — all of which were conducted within the European demographic. Moreover, nine independent genetic variants for Lp(a) levels, adhering to a linkage disequilibrium threshold of *R²* < 0.001 (as detailed in **Table 2**), were identified and employed as instrumental variables on the strength of a GWAS significance level of *p* < 5 × 10^-8. |
|  | b) | Report any additional statistics (e.g., assessments of heterogeneity across genetic variants, such as *I^2^*, Q statistic or E-value) | 5 | **Analysis of heterogeneity and horizontal pleiotropy**  In the current MR analysis, the modified Cochran *Q* statistic indicated an absence of significant heterogeneity (*p* > 0.05). Furthermore, the MR pleiotropic tests did not demonstrate any horizontal pleiotropy (intercept *p*-value > 0.05), implying that the results of our study are both robust and reliable. |
| 13 | **Sensitivity analyses and additional analyses** |  |  |  |
|  | a) | Report any sensitivity analyses to assess the robustness of the main results to violations of the assumptions | 5 | **Sensitivity analysis for MR analysis**  To ascertain the reliability of our methodology, we employed various validation methods, such as the weighted median and MR-Egger analysis. **Figures 2 and 3** depict that comparable causal estimations emerged from the sensitivity analysis, albeit with diminished precision. |
|  | b) | Report results from other sensitivity analyses or additional analyses | 5 | **Sensitivity analysis for MR analysis**  To ascertain the reliability of our methodology, we employed various validation methods, such as the weighted median and MR-Egger analysis. **Figures 2 and 3** depict that comparable causal estimations emerged from the sensitivity analysis, albeit with diminished precision. |
|  | c) | Report any assessment of direction of causal relationship (e.g., bidirectional MR) | - | - |
|  | d) | When relevant, report and compare with estimates from non-MR analyses | - | - |
|  | e) | Consider additional plots to visualize results (e.g., leave-one-out analyses) | 5 | **Sensitivity analysis for MR analysis**  To ascertain the reliability of our methodology, we employed various validation methods, such as the weighted median and MR-Egger analysis. **Figures 2 and 3** depict that comparable causal estimations emerged from the sensitivity analysis, albeit with diminished precision. |
|  | **DISCUSSION** |  |  |  |
| 14 | **Key results** | Summarize key results with reference to study objectives | 5 | In the current MR study, we explored the genetic causal relationships between Lp(a) levels and the risk of nine CVDs. Our findings revealed a causative association between elevated Lp(a)levels and an increased risk of CAD, AF, heart failure, hypertension, and PAD. Notably we also observed that genetically increased SA levels were significantly correlated with reduced Lp(a) levels, supporting the potential of aspirin as a therapeutic strategy to reduce the cardiovascular risk for individuals with elevated Lp(a) levels. |
| 15 | **Limitations** | Discuss limitations of the study, taking into account the validity of the IV assumptions, other sources of potential bias, and imprecision. Discuss both direction and magnitude of any potential bias and any efforts to address them |  |  |
| 16 | **Interpretation** |  |  |  |
|  | a) | Meaning: Give a cautious overall interpretation of results in the context of their limitations and in comparison with other studies | 8 | **Limitations** Several limitations should be acknowledged in this study. Primarily, due to reliance on summary data, individual patient-level information was inaccessible; consequently, it was not feasible to stratify the association between Lp(a) and CVDs by sex and age within this study. Second, the research encompassed solely the European demographic; hence, additional studies are requisite to determine whether these results are applicable to other ethnicities. Third, given the inherent limitations of MR studies, although we used MR-Egger, weighted median, and Cochran *Q* test to detect and reduce pleiotropy and heterogeneity, residual bias for undetected pleiotropy or gene-environment interactions could not be entirely ruled out. Fourth, multiple testing may increase the risk of type I error, thus some statistically significant findings should be interpreted with caution. Fifth, our results suggested that genetically predicted SA levels were inversely correlated with Lp (a), but SA is not unique to aspirin metabolism and lacks the pharmacological effects of aspirin. Therefore, our findings provide indirect evidence for the potential Lp(a)-lowering effects of aspirin, which still require further clinical validation in the future. Finally, the observed odds ratios for hypertension, peripheral artery disease, and non-rheumatic valve diseases were relatively modest, which may be influenced by the large sample size used in this study. Therefore, these findings should be interpreted with caution. Further research is needed to reaffirm the causal links between Lp(a) levels and these three cardiovascular conditions. |
|  | b) | Mechanism: Discuss underlying biological mechanisms that could drive a potential causal relationship between the investigated exposure and the outcome, and whether the gene-environment equivalence assumption is reasonable. Use causal language carefully, clarifying that IV estimates may provide causal effects only under certain assumptions | 5-7 | Growing evidence from observational studies supported the role of Lp(a) in atherogenesis and thrombosis [42-44]. The Copenhagen City Heart Study from the general Danish population examined 9330 individuals for 10 years and found that elevated Lp(a) levels were related to an increased risk of myocardial infarction (MI) [42]. A meta-analysis of 36 prospective studies summarized a total of 126,634 individuals indicated that there was a continuous and robust association between Lp(a) concentration and the risk of CAD [43]. Another analysis from the UK Biobank database enrolled 460,506 individuals demonstrated a linear relationship between Lp(a) and the risk of CAD during a median of 11.2 years follow-up [44]. Two large genetic epidemiological studies causally revealed Lp(a) concentration in the CAD population [12, 13]. Consistent with these findings, our MR analysis supports the causal relationship between elevated Lp (a) levels and an increased risk of CAD. Epidemiologic studies indicated only an extremely high Lp(a) concentration was correlated with a slightly increased risk of venous thromboembolism[45]. However, we did not observe a causal association between Lp(a) levels and DVT of lower extremities or PE in this study, which is consistent with a previous MR analysis [46].  Studies of evaluating the impact of Lp(a) levels on AF are limited and the relationship between the two has not been evaluated effectively. A community-based cohort study with a median follow-up of 13.9 years indicated that elevated Lp(a) levels were not correlated with the risk of AF [47]. However, this observational cohort study did not include extremely high Lp(a) concentrations and excluded individuals aged over 65 years old; the results may be influenced by potential confounders and sample selection bias. In contrast, a recent observational study conducted in the UK biobank database suggested each 50 nmol/L elevation in Lp(a) was correlated with an increased risk of AF occurrence [Hazard ratio (HR): 1.03; 95% CI: 1.02-1.04 ; p ＜0.05] [48]. Consistent with our study, the MR analyses revealed that Lp(a) may be a causal mediator in the incidence of AF [48]. A large prospective cohort from Europe demonstrated that Lp(a) levels were significantly associated with the occurrence of peripheral artery disease [49]. Our results by MR analysis further complemented the causal association between Lp(a) levels and the risk of PAD.  The MESA (Multi-Ethnic Study of Atherosclerosis) study, which included 6,809 participants, suggested that elevated Lp(a) levels were associated with a higher risk of heart failure [Lp(a) ≥50 mg/dL HR, 1.87; p=0.006] in the white population , which is consistence with our results [50]. Similarly, although limited data exists on the role of Lp (a) in hypertension, a cohort study found that 30% of hypertensive individuals had evaluated Lp(a) levels[51]. Despite these findings, observational studies alone cannot clarify the precise association between Lp(a) levels and the incidence of heart failure and hypertension. Our study is the first to demonstrate a causal relationship between elevated Lp (a) levels and the risk of both heart failure and hypertension.  The question of whether targeted Lp(a) reduction therapies can mitigate cardiovascular event risks has ignited considerable interest. However, the current challenge is the absence of specific therapies aimed at lowering Lp(a) levels. A randomized controlled trial (RCT) by Lacaze et al. examined the effect of aspirin on subjects with genotypes related to high plasma Lp (a), revealing that aspirin diminished the incidence of major adverse cardiovascular events (MACE) in individuals over 70 years old [22]. However, this RCT could not eliminate the potential impact of other variables, such as statin therapy, which may elevate Lp(a) levels. Furthermore, direct measurements of Lp(a) levels were not taken, leaving the specific influence of aspirin on Lp(a) levels undetermined. Notably, Bhatia et al. conducted an observational study using the MESA cohort and demonstrated that aspirin use was associated with a significantly lower risk of CHD events in individuals with Lp(a) > 50 mg/dL, suggesting a potential benefit of aspirin for primary prevention in this population[52]. However, their study was limited by the inherent drawbacks of observational studies, such as the potential bias, and the definition of aspirin use was based on patient self-reports. In contrast, our MR study leveraged genetic variants to minimize confounding and reverse causality, providing supportive causal inference regarding the inverse association between SA levels and Lp(a) concentrations, Increasing the possibility that aspirin may lead to a decrease in Lp (a). These studies underscore the urgent need for RCT to clarify the role of aspirin in managing cardiovascular risk associated with elevated Lp(a). To date, the mechanism by which aspirin may reduce Lp(a) levels remains unclear. Previous in vitro studies have shown that aspirin and sodium salicylate can inhibit the transcription and mRNA expression of apo(a) gene in human hepatocytes, thereby reducing Lp(a) synthesis, while the non-selective cyclooxygenase inhibitor indomethacin is ineffective, indicating that this process is independent of cyclooxygenase inhibition[17, 18]. These results are consistent with Mendelian randomization analyses that we found a negative correlation between SA levels and Lp(a) concentrations. Further studies are needed to clarify this mechanism and its clinical implications. |
|  | c) | Clinical relevance: Discuss whether the results have clinical or public policy relevance, and to what extent they inform effect sizes of possible interventions | 7 | The principal merit of this investigation lies in the application of the MR methodology to establish a causal relationship between Lp(a) levels and the risks of nine CVDs within a relatively homogeneous population, distinctly without overlap in the study cohort. Our findings bolster the evidence for a causal link between Lp (a) and cardiovascular risk, aligning with the recent declaration by the European Atherosclerosis Society consensus [6]. |
| 17 | **Generalizability** | Discuss the generalizability of the study results (a) to other populations, (b) across other exposure periods/timings, and (c) across other levels of exposure | 8 | Second, the research encompassed solely the European demographic; hence, additional studies are requisite to determine whether these results are applicable to other ethnicities. |
|  | **OTHER INFORMATION** |  |  |  |
| 18 | **Funding** | Describe sources of funding and the role of funders in the present study and, if applicable, sources of funding for the databases and original study or studies on which the present study is based | 8 | This study was supported by National Clinical Medical Research Center for Cardiovascular Diseases (NCRC2020015), High-Level Hospital Clinical Research Funding (2022-GSP-GG-26), and Fundamental Research Funds for the Central Universities (2024-XHQN15). |
| 19 | **Data and data sharing** | Provide the data used to perform all analyses or report where and how the data can be accessed, and reference these sources in the article. Provide the statistical code needed to reproduce the results in the article, or report whether the code is publicly accessible and if so, where | 8 | **Availability of Data and Materials**  All summary-level GWAS data used in this study are publicly available from the IEU OpenGWAS database (https://gwas.mrcieu.ac.uk/). No individual-level data were used. : The analysis code is available upon reasonable request from the corresponding author. |
| 20 | **Conflicts of Interest** | All authors should declare all potential conflicts of interest | 8 | **Conflict of Interest**  The authors declare no conflict of interest. |

This checklist is copyrighted by the Equator Network under the Creative Commons Attribution 3.0 Unported (CC BY 3.0) license.

1. Skrivankova VW, Richmond RC, Woolf BAR, Yarmolinsky J, Davies NM, Swanson SA, et al. Strengthening the Reporting of Observational Studies in Epidemiology using Mendelian Randomization (STROBE-MR) Statement. JAMA. 2021;under review.

2. Skrivankova VW, Richmond RC, Woolf BAR, Davies NM, Swanson SA, VanderWeele TJ, et al. Strengthening the Reporting of Observational Studies in Epidemiology using Mendelian Randomisation (STROBE-MR): Explanation and Elaboration. BMJ. 2021;375:n2233.
